# Drug-Online: an online platform for drug-target interaction, affinity, and binding sites identification using deep learning

**DOI:** 10.1186/s12859-024-05783-w

**Published:** 2024-04-20

**Authors:** Xin Zeng, Guang-Peng Su, Shu-Juan Li, Shuang-Qing Lv, Meng-Liang Wen, Yi Li

**Affiliations:** 1 College of Mathematics and Computer Science, Dali University, Dali, 671003 China; 2 Yunnan Institute of Endemic Diseases Control and Prevention, Dali, 671000 China; 3 Institute of Surveying and Information Engineering West, Yunnan University of Applied Science, Dali, 671000 China; 4 State Key Laboratory for Conservation and Utilization of Bio-Resources in Yunnan, Yunnan University, Kunming, 650000 China

**Keywords:** Online platform, Deep learning, Drug-target interaction, Drug-target affinity, Drug-target binding sites

## Abstract

**Background:**

Accurately identifying drug-target interaction (DTI), affinity (DTA), and binding sites (DTS) is crucial for drug screening, repositioning, and design, as well as for understanding the functions of target. Although there are a few online platforms based on deep learning for drug-target interaction, affinity, and binding sites identification, there is currently no integrated online platforms for all three aspects.

**Results:**

Our solution, the novel integrated online platform Drug-Online, has been developed to facilitate drug screening, target identification, and understanding the functions of target in a progressive manner of “interaction-affinity-binding sites”. Drug-Online platform consists of three parts: the first part uses the drug-target interaction identification method MGraphDTA, based on graph neural networks (GNN) and convolutional neural networks (CNN), to identify whether there is a drug-target interaction. If an interaction is identified, the second part employs the drug-target affinity identification method MMDTA, also based on GNN and CNN, to calculate the strength of drug-target interaction, i.e., affinity. Finally, the third part identifies drug-target binding sites, i.e., pockets. The method pt-lm-gnn used in this part is also based on GNN.

**Conclusions:**

Drug-Online is a reliable online platform that integrates drug-target interaction, affinity, and binding sites identification. It is freely available via the Internet at http://39.106.7.26:8000/Drug-Online/.

## Background

Accurately identifying drug-target interaction (DTI), affinity (DTA), and binding sites (DTS) through deep learning techniques is essential for various aspects of drug research, including screening, repositioning, design, and target identification from massive data. It also provides valuable insights into the functions of target. Drug-target interaction identification calculates whether a given drug and target interact, which is a prerequisite for their reaction [1]. Affinity measures the interaction strength between drug and target, with drug having strong binding affinity used as candidate for biological experimental verification [2]. However, any cavities on the surface of target may also become binding sites (pockets) for drug. Identifying drug-target binding sites has become a critical factor in drug screening and design, further narrowing the scope of biological experimental verification [3]. Thus, integrating drug-target interaction, affinity, and binding sites identification forms an online screening platform for achieving precise drug design, narrowing the scope of drug screening and biological experimental verification, shortening the drug development cycle, and promoting the rapid development of the modern pharmaceutical industry.

Currently, most computational-based methods for identifying drug-target interaction, affinity, and binding sites [4–12] utilize deep learning techniques. The general process of these deep learning-based methods involves first collecting a dataset of drug-target interaction. Then, deep learning models such as graph neural networks (GNN) [13], convolutional neural networks (CNN) [14], recurrent neural networks (RNN) [15], and attention mechanisms [16] are employed to extract high-level hidden features from the sequences, structures, or complexes of drugs and targets. Finally, based on the extracted features, a fully connected (FC) network is used to identify drug-target interaction, affinity, and binding sites.

Next, firstly let us consider some examples of drug-target interaction identification methods. DeepLPI method [17] focused solely on drug SMILES (Simplified Molecular Input Line Entry System) [18] and target sequences. It employed a ResNet-based 1-dimensional convolutional neural network (1D-CNN) to extract sequence features from drug and target. These sequence features were then fused using a bi-directional long short term memory network (biLSTM) and inputed into a FC network to predict drug-target interaction. On the other hand, DeepMGT-DTI method [19] converted drug SMILES into the molecular graphs and utilized molecular complementary graph convolutional neural networks (MCGCN) and Transformers to extract high-level features from drug molecular structures. Additionally, it employed CNN to extract high-level features from target sequences. By concatenating the extracted features, the FC network predicted the presence or absence of drug-target interaction. Another approach, described in reference [20], involved using two graph convolutional networks (GCNs) to extract structural features. One network processed drug-target binding pocket graphs, while the other handled 2D drug molecule graphs. These features were then used to predict drug-target interaction. Secondly, for deep learning-based drug-target affinity prediction methods, both DeepDTA [21] and DeepDTAF [22] used 1D-CNN to extract sequence features from drugs and targets for predicting drug-target affinity. GraphDTA [23], SAG-DTA [24], GraphCL-DTA [25], TDGraphDTA [26], and other related methods employed GNN to extract the structural features of drug molecules. Meanwhile, natural language processing models such as CNN and RNN were utilized to extract sequence features. Furthermore, method proposed by [27] and MMDTA [28] simultaneously made use of both the sequence and structural features of drug and target. Finally, deep learning-based methods for predicting drug-target binding sites follow a similar approach to the feature extraction methods mentioned above for drug-target interaction and affinity prediction. One such method was the DeepProSite [3] approach. It began by constructing a graph based on the structure of target and extracting the sequence features from target sequence. These features were used as node features in the graph of target, obtained through a pre-trained model. Graph Transformer was then used to extract structural features from the graph of target. Subsequently, a multi-layer perceptron (MLP) was used to predict whether the nodes in the graph corresponded to binding sites. Another method, SiteRadar [29], utilized graph machine learning to accurately predict the binding pockets (sites) between drugs and targets.

Although deep learning-based methods for drug-target interaction, affinity, and binding sites identification have provided complete datasets and codes, only a few have made online platforms available for biologists and pharmacists to use [30, 31]. However, it can be challenging for biologists and pharmacists to reproduce these codes and run them on the provided datasets. Therefore, developing an online platform directly for biologists and pharmacists based on deep learning methods for drug-target interaction, affinity, and binding sites identification can truly promote the practical applications of computational-based research results about drug-target interaction, affinity, and binding sites identification.

In this study, we developed an integrated online platform based on deep learning for drug-target interaction, affinity, and binding sites identification, called Drug-Online. Drug-Online platform consisted of three parts: the first part was to identify whether there was interaction between drug-target pair, using the MGraphDTA [32] method based on GNN and CNN. If there was an interaction between the drug-target pair tested in the first part, continued to calculate the binding affinity of drug-target pair in the second part, using the multimodal method MMDTA based on GNN and CNN. In the third part, pt-lm-gnn method [33] was directly used to identify the specific binding sites of drug-target pair with strong binding affinity. Drug-Online was available for free online use by biologists and pharmacists, providing assistance in narrowing the scope of biological experiments required for screening drug molecules, identifying targets, and analyzing the functions of target.

## Implementation

### Datasets

In this study, we initially approached drug-target interaction identification as a binary classification task using the MGraphDTA method [32], leveraging two widely used classification datasets: Human and Caenorhabditis elegans (C.elegans) [34–36]. Subsequently, we regarded drug-target affinity identification as a regression task employing the MMDTA method [28], which was trained and tested on the PDBbind dataset (version 2016) [37]. Finally, we tackled drug-target binding sites identification as a classification task using the pt-lm-gnn method [33], making use of a standard dataset developed by Yu et al. [38].

### Methods

Drug-Online platform for identifying drug-target interaction, affinity, and binding sites comprises three deep learning-based methods: the MGraphDTA method [32] for identifying drug-target interaction, the MMDTA method [28] for predicting drug-target affinity, and the pt-lm-gnn method [33] for identifying drug-target binding sites. Next, we will provide a brief overview of the architectures of these three methods.

#### MGraphDTA

To begin, drug SMILES was first processed into the molecular graphs using RDKit tool [39]. Subsequently, multi-scale graph neural network (MGNN) was employed to extract the structural features of drugs, and the extracted structural features were fused. With regards to the targets, multi-scale convolutional neural network (MCNN) was utilized to extract the sequence features from target sequences, and these features were fused as well. Next, the fused structural features of drugs and the fused sequence features of targets were concatenated to obtain the combined features of drug-target pair. Finally, the combined features of drug-target pair were input into MLP to predict drug-target interaction.

#### MMDTA

MMDTA was a deep learning-based approach for predicting drug-target affinity using multimodal information from both drugs and targets. GNN and CNN were utilized to extract the sequence and structural features from the SMILES and 2D molecular graphs of drugs, respectively. For target, its sequence features were extracted through 1D-CNN. As for its structural features, the distance matrix was first constructed based on the three-dimensional (3D) structure of target, followed by obtaining its structural features through a 2-dimensional convolutional neural network (2D-CNN). The extracted sequence and structural features of drugs and targets were then input into a hybrid fusion module to comprehensively integrate and fuse the interaction information between drugs and targets. Subsequently, the fused features were fed into a FC network to predict drug-target affinity.

#### pt-lm-gnn

Pt-lm-gnn method was a hybrid model that combined target language model with GNN to predict drug-target binding sites. The target language model was utilized to extract sequence features from target sequences. Additionally, a target graph was constructed based on the 3D structure of target, where amino acid residues served as nodes in the graph. Simultaneously, the sequence features of target were employed as node features. Graph attention network (GAT) was then applied to extract deep structural features of target, enabling node classification to acquire drug-target binding sites.

## Results

**Fig. 1:**
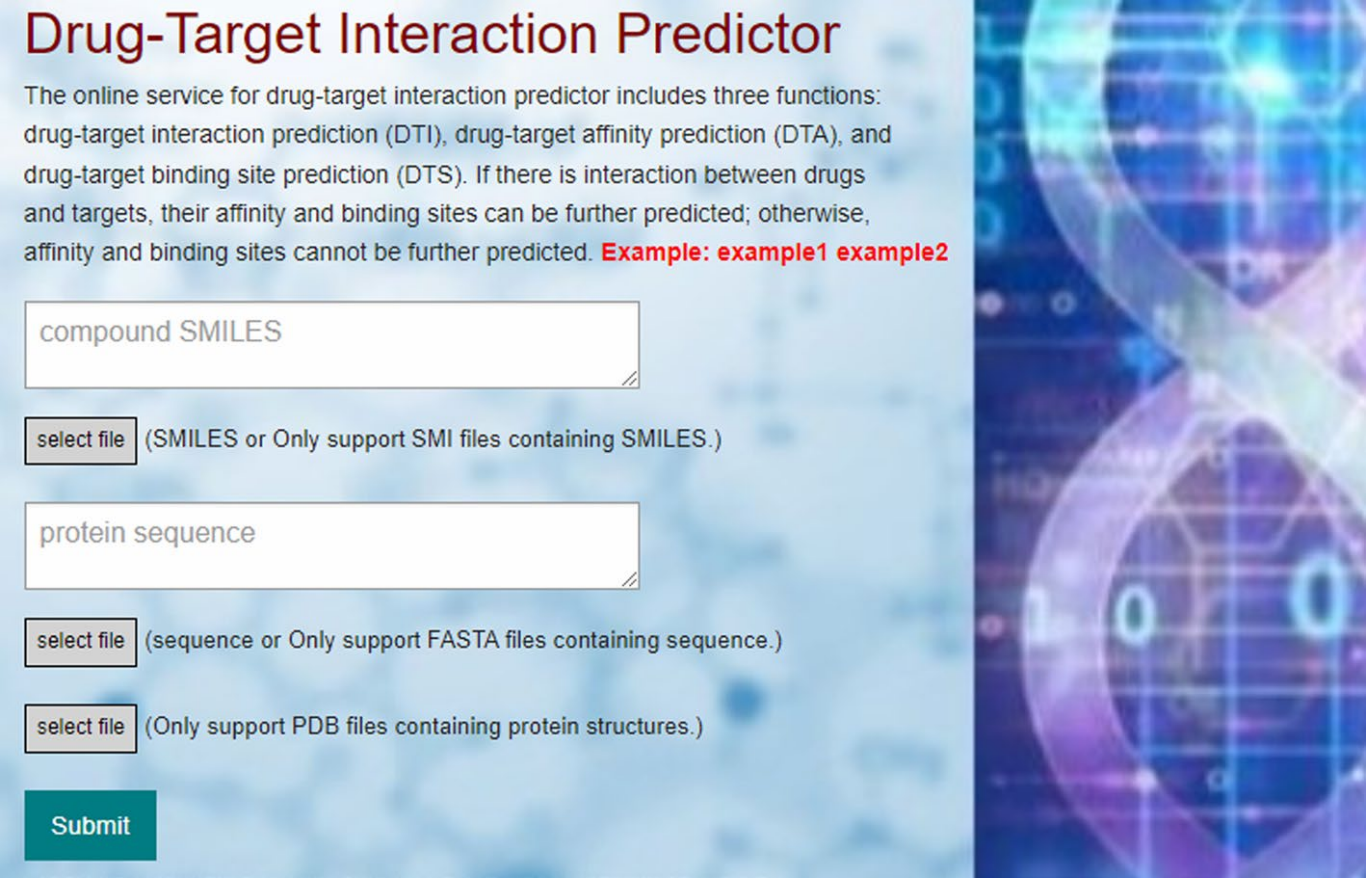
The input interface of Drug-Online. We need to provide drug SMILES (compound SMILES), target sequence (protein sequence), and structure (PDB file). Drug SMILES and target sequence can be directly entered into the respective text boxes, or their corresponding files (.smi for drug SMILES and.fasta for target sequence) can be uploaded using the file upload button. The PDB structural file of target can only be uploaded using the file upload function

Our developed online platform Drug-Online for drug-target interaction, affinity, and binding sites identification comprised three main components (Fig. 1): TOOLS, API (Application Programming Interface), and HELP. The TOOLS section is designed for identifying drug-target interaction, affinity, and binding sites. The API section offers detailed instructions for other programs to access Drug-Online for their own use. The HELP section provides comprehensive information, usage guidelines, and solutions to any issues that may arise while using Drug-Online.

### Input

Drug-Online platform offers three primary functions: drug-target interaction, affinity, and binding sites identification. In the input interface (Fig. 1), we need to provide drug SMILES (compound SMILES), target sequence (protein sequence), and structure (PDB file). During the operation, drug SMILES and target sequence can be directly entered into the respective text boxes, or their corresponding files (.smi for drug SMILES and fasta for target sequence) can be uploaded using the file upload button. However, the PDB structural file of target can only be uploaded using the file upload function. The input target structural file can be the 3D structure of target obtained from the PDB database or it can be a prediction tool such as AlphaFold [40] and ColabFold [41] for predicting the structure of target. The required drug molecular graph is generated by converting drug SMILES using the built-in RDKit tool.

### Output

**Fig. 2:**
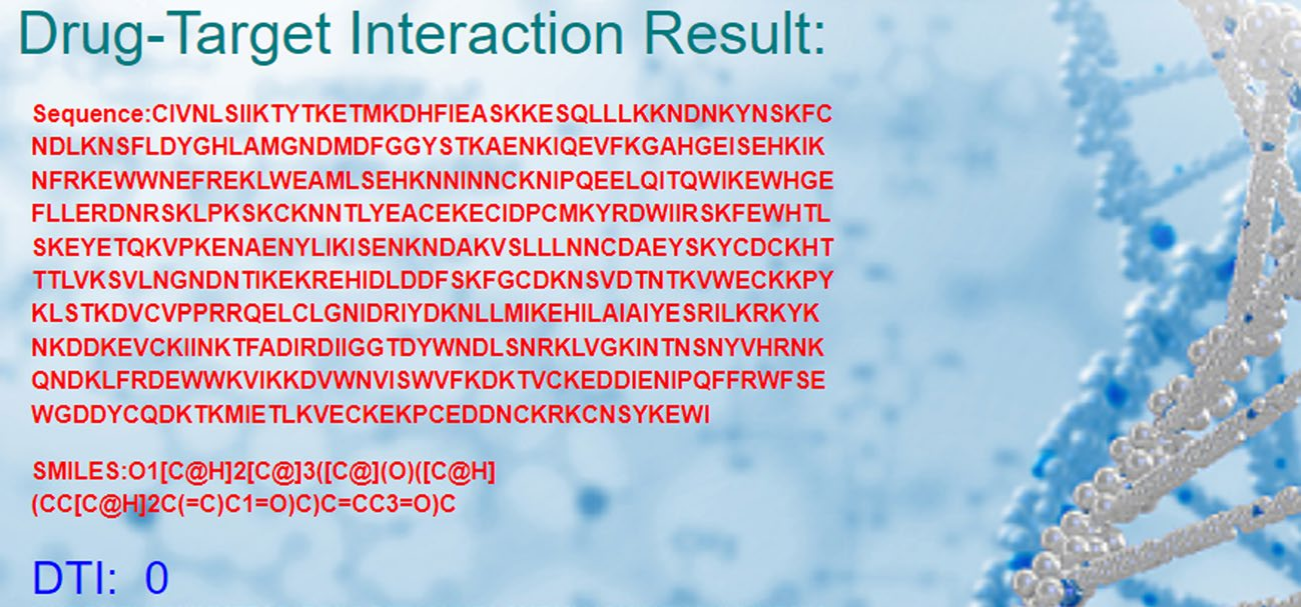
The output result interface where there is no interaction between drug-target pair. Because there is no interaction between the input drug and target, the output value of DTI is 0 (indicating no interaction)

The output from Drug-Online platform yields two possible results: 1) If there is no interaction between the input drug and target, the output value of DTI is 0 (indicating no interaction), and the affinity and binding sites identification programs are not further executed by Drug-Online. For example, if drug SMILES file is 442288.smi, target sequence file is A0A023ZZ89.fasta, and target structural file is AF-A0A023ZZ89-F1-model_v4.pdb, the resulting output of 0 indicates that there is no interaction between the input drug and target (Fig. 2).

**Fig. 3:**
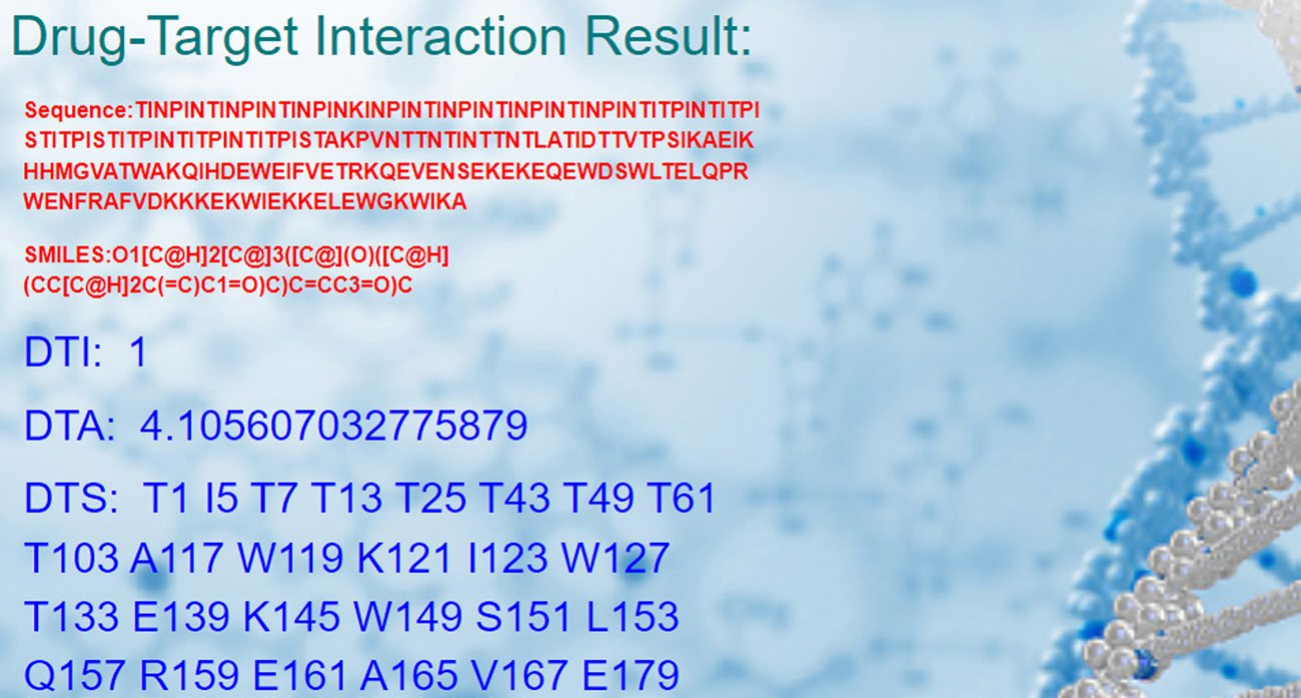
The output result interface where there is an interaction between drug-target pair. Because there is an interaction between the input drug and target, in addition to the output value of DTI is 1 (indicating interaction), Drug-Online will proceed to run the affinity (DTA) and binding sites (DTS) identification programs and provide the calculation results

2) If there is an interaction between the input drug and target, in addition to the output value of DTI is 1 (indicating interaction), Drug-Online will proceed to run the affinity and binding sites identification programs and provide the calculation results. For instance, if drug SMILES file is 442288.smi, target sequence file is A0A0A1DGA0.fasta, and target structural file is AF-A0A1DGA0-F1-model_v4.pdb, the output results consist of three parts: the drug-target interaction (DTI) result is 1, the drug-target affinity (DTA) result is 4.105607032775879, and the drug-target binding sites (DTS) is identified as T1 I5 T7 T13 T25 T43 T49 T61 T103 A117 W119 K121 I123 W127 T133 E139 K145 W149 S151 L153 Q157 R159 E161 A165 V167 E179 (Fig. 3).

### API and HELP

In addition to offering online applications for drug-target interaction, affinity, and binding sites identification, Drug-Online also provides the API function, enabling users to access these three identification tools through other programs. To enhance user experience, we have included a comprehensive explanation of the Drug-Online platform in the HELP section. Furthermore, we have presented a detailed case study that demonstrates step-by-step usage of Drug-Online platform, ensuring a smooth experience for biologists, pharmacologists, and other researchers in the field.

## Conclusions

Currently, most state-of-the-art deep learning methods used in drug development, such as drug-target interaction, affinity, and binding sites identification, only offer datasets and codes, with a few providing online platforms directly accessible to biologists and pharmacologists. This limits the application and dissemination of advanced computational-based drug screening methods and makes it challenging to translate the research achievements into practical applications. While our developed Drug-Online platform offers drug-target interaction, affinity, and binding sites identification applications for biologists and pharmacologists, it also has some limitations:

1. Deep learning-based methods employed by Drug-Online platform primarily rely on GNN models, resulting in a relatively singular architecture. This restricts our ability to provide users with diverse combinations methods tailored to different focuses.
2. Deep learning-based methods utilized by Drug-Online platform were trained and tested on different datasets, potentially leading to variations in performance when confronted with different input data.

Moving forward, we aim not only to encourage researchers studying computational methods related to drug screening to develop their own online platforms based on their research findings, but also to address the shortcomings of Drug-Online. Our objective is to create a fully functional and high-performance integrated online platform, which truly offers user-friendly services for biologists and pharmacists in the field of computational-based drug screening. By enabling the swift translation of advanced research outcomes into practical application tools, we strive to expand the scope of application for these research results.

## Data Availability

Drug-Online platform can be accessed at http://39.106.7.26:8000/Drug-Online/. Project name: Drug-Online. Home page: http://39.106.7.26:8000/Drug-Online/. Operating system(s): Platform independent. Programming language: Python. Other requirements: Web browser. License: BSD 3-Clause License. Any restrictions to use by non-academics: None.

## Abbreviations

DTI: Drug-target interaction
DTA: Drug-target affinity
DTS: Drug-target binding sites
GNN: Graph neural networks
CNN: Convolutional neural networks
RNN: Recurrent neural networks
FC: Fully connected
1D-CNN: 1-Dimensional convolutional neural network
biLSTM: Bi-directional long short term memory network
MCGCN: Molecular complementary graph convolutional neural networks
GCNs: Graph convolutional networks
MLP: Multi-layer perceptron
MGNN: Multi-scale graph neural network
MCNN: Multi-scale convolutional neural network
2D-CNN: 2-Dimensional convolutional neural network
GAT: Graph attention network
